# Association of dietary calcium intake, total and ionized serum calcium levels with preeclampsia in Ethiopia

**DOI:** 10.1186/s12884-021-04005-y

**Published:** 2021-07-27

**Authors:** Rahel D. Gebreyohannes, Ahmed Abdella, Wondimu Ayele, Ahizechukwu C. Eke

**Affiliations:** 1 Department of Obstetrics and Gynecology, Addis Ababa University, College of Health Sciences, Addis Ababa, Ethiopia; 2 Department of Preventive Medicine, Addis Ababa University, School of Public Health, Addis Ababa, Ethiopia; 3 Division of Maternal Fetal Medicine, Department of Gynecology and Obstetrics, Johns Hopkins University School of Medicine, Baltimore, MD USA

**Keywords:** Pre-eclampsia, Total serum calcium level, Dietary calcium, Ionized serum calcium level

## Abstract

**Background:**

Preeclampsia is a well-known cause of maternal mortality and morbidity in Ethiopia. The exact pathophysiology has not been fully understood. Calcium and magnesium deficiencies have been given emphasis to play roles in the pathophysiology. Although evidence is abundant, they are equivocal. The study aimed to see the association of dietary calcium intake, serum total calcium level and ionized calcium level with preeclampsia. It also evaluated the association between dietary calcium intake and serum calcium levels.

**Materials and methods:**

An unmatched case–control study was conducted in Gandhi Memorial, Tikur Anbessa, and Zewditu Memorial Hospitals, all in Addis Ababa, between October to December, 2019. Cases were 42 women with preeclampsia and controls were 42 normotensive women. The medical and obstetric history was gathered using a structured questionnaire and the dietary calcium intake information using a 24-h dietary recall. The serum levels of total serum calcium and ionized (free) calcium were measured using an inductively coupled mass spectrophotometer. Bivariate and multivariate logistic regression and Pearson correlation test were utilized during data analysis.

**Results:**

In comparison with controls, women with preeclampsia had lower mean (± 1SD) levels of ionized calcium level (1.1 mmol/l ± 0.11), total serum calcium level (1.99 mmol/l ± 0.35) and lower median (IQR) dietary calcium intake (704 mg/24 h,458–1183). The odds of having preeclampsia was almost eight times greater in those participants with low serum ionized calcium level (OR 7.5, 95% CI 2.388–23.608) and three times higher in those with low total serum calcium level (OR 3.0, 95% CI 1.024–9.370). Low dietary calcium intake also showed statistically significant association with preeclampsia (OR 3.4, 95% CI 1.092 -10.723). Serum ionized calcium level and total serum calcium level showed positive correlation of moderate strength (*p* = 0.004, *r* = 0.307), but no correlation was found between dietary calcium intake with both forms of serum calcium levels.

**Conclusion:**

This study showed significant association between low dietary calcium intake and low serum calcium levels with preeclampsia, hence this can be used as a supportive local evidence for the current context-specific recommendation of calcium supplementation in societies with low-dietary calcium consumption in an attempt to prevent preeclampsia, therefore implementation study should be considered in Ethiopia to look for the feasibility of routine supplementation.

**Supplementary Information:**

The online version contains supplementary material available at 10.1186/s12884-021-04005-y.

## Background

High blood pressure (with or without proteinuria) is a major cause of maternal and perinatal morbidity and mortality worldwide [1, 2]. Preeclampsia complicates about 5–10% of all pregnancies and accounts for > 50,000 maternal deaths each year [2, 3]. In studies done in different parts of Ethiopia including the capital Addis Ababa, prevalence ranged between 7.2% and 8.4% [4–8].

The etiology of this condition remains unknown, although believed to be multifactorial. Calcium deficiency is among the etiologies that have been implicated [9]. Currently, there is an increased interest in the management and prevention of preeclampsia with nutritional supplements, such as the use of calcium and magnesium supplementation. However, reports from clinical studies are inconsistent on the role of calcium and magnesium in the etiology and prevention of preeclampsia. Reports from different countries such as India, Sudan, South Korea, and Iran have shown significant relationships between low serum calcium levels and preeclampsia [10–13], whereas, other studies from Ghana, Nigeria, South Africa and India have shown no difference in serum calcium levels in hypertensive and normotensive pregnant women [14–16].

The decrease in dietary calcium intake will decrease serum calcium concentrations, thereby inducing the release of parathyroid hormone, which in-turn increases the intracellular calcium levels, causing vasoconstriction [17]. But there are several evidences that the serum level of calcium does not solely depend on dietary intake of calcium. A study in India reported the association of dietary calcium intake, serum calcium level and preeclampsia, and noted that study subjects had normal serum calcium level despite very low intake of dietary calcium during pregnancy. The possible explanation for their finding was the possibility of greater exposure of the study participants to sunlight and increased vitamin D production. This may be possible as the serum calcium levels are tightly regulated by hormones (Parathyroid hormone and Calcitonin) and the prohormone Vitamin D and through organs (bone, kidney and small intestine) through complex feedback loops [17, 18].

About half of the plasma calcium is found in ionized (free) form and the rest is bound to protein (40%) and anions. The ionized calcium is the fraction that is biologically active and tightly regulated by hormones. During pregnancy, the ionized calcium remains constant throughout gestation, but the total calcium concentration varies significantly due to hemodilution [18]. It is therefore evident that serum ionized calcium is by far the most important index to know the actual calcium status of the body compared to the total serum calcium level.

World Health Organization (WHO) currently recommends calcium supplementation at a dose of 1.5–2 g/day starting in the first half of pregnancy for prevention of preeclampsia for those populations with generally low dietary calcium intake. However, the recommendation for calcium supplementation is still context specific because the implementation clearly may not be easy considering the cost and burden of the drugs to incorporate the recommendation in to the local guideline [19].

Typically, daily calcium intake in low-income countries is 300-600 mg/day compared with 855 mg/day in the United Kingdom and 969 mg/day in France [20]. A big National Survey has been done to assess the dietary calcium intake of the average Ethiopian women in the child bearing age, and demonstrated that dietary calcium intake of women in this age group was very low (i.e. 317.32 mg/day) compared with the recommended daily allowance (RDA) [21]. The RDA should be at least 1,000 mg/day for pregnant women. With all the evidences of low dietary calcium intake and low serum calcium levels in the Ethiopian community, no study has been done to demonstrate any associations between serum calcium concentrations (both total and ionized) and dietary calcium intake and the development of pre-eclampsia in Ethiopian women, prompting the research gap filled by this study. Hence, this study aimed to see the association of dietary calcium intake, serum total calcium level and ionized calcium level with preeclampsia. It also evaluated the correlation between dietary calcium intake and serum calcium levels and the correlation between serum total calcium level and ionized calcium level.

## Methods

### Study design

Unmatched case–control study was done at three big hospitals in the capital Addis Ababa: Gandhi Memorial Hospital, Tikur Anbessa Hospital and Zewditu Memorial Hospital. The study was completed in 84 participants (42 cases and 42 controls). The required sample size was determined using unmatched case control study sample size calculation formula. Given the lack of concrete information from previous studies on the prevalence of low dietary calcium intake and low serum calcium level among preeclamptic women in similar setting, in order to get representative sample size, we assumed hypothetically identified odds ratio of 4.0 that could yield the representative sample size, a confidence level of 95% and power of 80%. Adjustment was made for population size (less than 10 times from estimated sample size) using finite population correction factor.

### Data collection procedures

Cases were recruited based on the current (2019) International Society for the Study of Hypertension in Pregnancy (ISSHP) definition of preeclampsia which is, systolic blood pressure at ≥ 140 mmHg and/or diastolic blood pressure at ≥ 90 mmHg on at least two occasions measured 4 hours apart in previously normotensive women and is accompanied by one or more of the following new onset conditions at or after 20 weeks of gestation: proteinuria (i.e. ≥ 30 mg/mol protein: creatinine ratio; ≥ 300 mg/24 h; or ≥ 2 + dipstick); Evidence of other maternal organ dysfunction, including: acute kidney injury (creatinine ≥ 90 μmol/L; 1 mg/dL); liver involvement (elevated transaminases, e.g. alanine aminotransferase or aspartate aminotransferase > 40 IU/L) with or without right upper quadrant or epigastric abdominal pain; neurological complications (e.g. eclampsia, altered mental status, blindness, stroke, clonus, severe headaches, and persistent visual scotomata); or hematological complications (thrombocytopenia–platelet count < 150 000/μl, disseminated intravascular coagulation, hemolysis); or uteroplacental dysfunction (such as fetal growth restriction, abnormal umbilical artery doppler waveform analysis, or stillbirth) [22]. Cases were selected from the antenatal clinics (ANC), but sometimes followed to the antepartum ward for repeat blood pressure (BP) measurement. Incident cases were chosen to decrease recall bias.

Inclusion criteria for the cases were women aged ≥ 18 years, gestational age ≥ 20 weeks and singleton pregnancy. Those with chronic hypertension, women on anti-hypertensive and anti-convulsant treatment, diabetes mellitus, renal disease, known thyroid and adrenal disease, intrauterine fetal death and those taking antenatal vitamin and mineral supplementation were excluded.

Controls were selected from the hospitals too (source population). Recruiting neighborhood and population controls was not possible in our setting, as the likelihood of finding a healthy non-hypertensive pregnant woman in the neighborhood was unlikely. Any risk factors that could contribute for preeclampsia in the current or previous pregnancies, such as antepartum hemorrhage (APH) and twin pregnancies, were excluded. Controls were women who came for normal ANC follow up, or with obstetric conditions like previous cesarean section scar, or women that could otherwise follow pregnancy at the health centers.

Systematic random sampling technique was used to recruit participants. With the assumption that there could be around 250 cases of preeclampsia in the data collection period, we recruited every sixth patient with preeclampsia and a corresponding number of controls was selected.

Recruitment of cases and controls and coding were done by the principal investigator and the treating physician at ANC and emergency room. Data collectors, who were nurses with Bachelor of Science (BSC) degree, were masked before they took the dietary recall, then unmasked after completing the collection of data related to the dietary recall, as information from patient cards was mandatory to complete the questionnaire. The diagnosis of preeclampsia was made by the consultation physician, and evidences were carefully recorded. Gestational age was determined with the most accurate obstetric estimate, using menstrual history, symphysis–fundal height at the initial antenatal (booking) visit or ultrasound scan, when available. The questionnaire comprised demographic details, booking details of the index pregnancy, general medical and health details, a dietary questionnaire. Blood pressure was measured by trained personnel using a manual pneumatic mercury sphygmomanometer, and utilizing the fifth Korotkoff sound. A repeat measurement was recorded after 4 h to ensure consistency in blood pressure measurement.

Ten milliliters of blood from antecubital fossa was taken, centrifuged, and refrigerated at 2–8 degree centigrade until analyzed. Total serum calcium and ionized serum calcium were determined by using an inductively coupled plasma mass spectrometer. Twenty-four-hour dietary recall was done and calcium content in the meals of the participants were calculated using the food composition table for use in Ethiopia, Part III, developed in 1995–1997 [23].

Data was collected from October 3 to December 25, 2019.

### Data analysis

Data was analyzed using SPSS version 25 (IBM, Armonk, NY, USA). Descriptive analyses, Chi-square test, univariate and multivariate logistic regressions were done. Odds ratios (OR), adjusted OR and 95% confidence intervals (CI) were calculated. Pearson/Spearman correlation was performed. *P* value of less than 0.05 was considered statistically significant. We used t-test statistic to see the association of the variables age, parity, gestational age and body mass index with preeclampsia. The association between dietary calcium intake and total and ionized serum calcium levels, educational level, body mass index, parity, age group and gestational age, income was seen using Chi-square test. Bivariate logistic regression was used to measure the relationship of each covariate with the outcome variable (preeclampsia). Variables that were associated with the outcome variable with *p*-value of < 0.2 in the univariate analysis and clinically relevant variables that were known to associate with preeclampsia despite their *p*-values in the univariate analysis were included in to the multiple logistic regression model. Hence, the variables imputed in the multiple logistic regression model were parity, gestational age, total serum calcium level, ionized calcium level and dietary calcium amount. Pearson correlation was used to see if dietary calcium intake, total serum calcium level and ionized calcium level correlated with each other.

Reference (normal) values taken were 2.1–2.55 mmol/l for total serum calcium level, 1.16–1.32 mmol/l for ionized calcium level and Recommended Dietary Allowance (RDA) of ≥ 1000 mg/24 h for dietary calcium intake.

## Results

Majority of the cases (n = 33, 78.6%), had preeclampsia with severe features. The remaining 9 cases (21.4%) had preeclampsia without severity features. Severe hypertension (n = 25, 60%) and other severity symptoms (n = 26, 62%) of preeclampsia were equally common. Six patients (18.2%) had HELLP syndrome (hemolysis, elevated liver enzymes, low platelets). Two of the cases (6%) had acute kidney injury, four (9.5%) had intrauterine growth restriction (IUGR), and one had eclampsia.

Most of the controls (n = 14, 57.1%) had low risk pregnancies, while the remaining controls had scarred uterus (n = 11, 26.2%), post-term pregnancies (n = 4, 9.5%), malpresentations (n = 2, 4.8%), and premature rupture of membranes (PROM) (n = 1,2.4%).

Nineteen percent (n = 8) of the cases and 38.1% (n = 16) of the controls had gone to the university. Majority (n = 24, 57.1%) of the controls and 40.4% (n = 17) of cases had gone to secondary school or beyond, the illiteracy rate of the women in both groups was low. The median per capita income of the cases was 411 USD (IQR 308–616) and the controls’ was 616 USD (IQR 411–821). Both income medians were below the GDP (2019) per capita income of Ethiopia [24]. Regarding employment, 52.4% (n = 22) of the controls and 59.5% (n = 25) of the cases were unemployed.

Both groups were comparable in their obstetric characteristics (age, parity, body mass index/BMI) except the mean gestational age (GA) of the controls (mean ± 1SD = 38.50 ± 3.64) was significantly higher (*p* = 0.04) than that of the cases (mean ± 1SD = 36.68 ± 3.83) (Table 1).

**Table 1:** Comparison of the means (sd) of obstetric characteristics of the case and control groups

*Variable*	*Cases (N* = *42)*		*Controls (N* = *42)*		*P*
	*Mean*	*SD*	*Mean*	*SD*	
Age (years)	27	6	27	4	0.824
GA (weeks)	36.68	3.83	38.50	3.64	0.04
Parity	0.73	1.05	1.04	1.68	0.083
Pre-pregnancy BMI (kg/m^2^)	23.29	3.25	23.22	4.06	0.63
Pregnancy BMI (Kg/m^2^)	26.20	3.47	26.15	4.21	0.897

*GA* Gestational age, *BMI* Body mass index, *SD* Standard deviation, *N* Number

The mean (± 1SD) total serum calcium level and ionized calcium level in the preeclamptic women was found to be in the hypocalcemia range—lower than the serum calcium concentration in the control groups. Twenty-nine (nearly 70%) of the cases and n = 15(35.7%) of the controls had low total serum calcium concentrations. Twenty-five (around 60%) controls had normal level of total serum calcium level whereas, only n = 11 (26%) of the women with preeclampsia had their total calcium level in the normal range (Table 2).

**Table 2:** Comparison of the means/medians of dietary calcium intake, total serum calcium level and ionized calcium levels in case and control groups

*Variable*	*Cases* *(n* = *42)*	*Controls* *(n* = *42)*	*P*
Total serum calcium level (mmol/l), (mean ± SD)	1.99 ± 0.35	2.19 ± 0.22	0.007
Ionized calcium level (mmol/l), (mean ± SD)	1.1 ± 0.11	1.2 ± 0.13	0.000
Dietary calcium (gram/24 h), (median, IQR)	704, 458–1183	1115,753–1878	0.049

*SD* Standard deviation, *IQR* Interquartile range, *n* Number

Majority of the cases (n = 29,69%) were found to have low ionized calcium level, whereas 9 of the controls (21.4%) had serum ionized calcium level below the reference range.

The median dietary calcium intake in the cases was lower than those in the controls and the normal range. Almost half of the controls (n = 24, 51%) had a dietary calcium intake equal to or more than the RDA, whereas only one third (n = 15, 35%) of the cases had an adequate dietary calcium intake (Table 2).

Univariate logistic regression showed that serum ionized calcium level and total serum calcium level showed statistically significant association with preeclampsia. Having low levels of both types of serum calcium levels is a risk for preeclampsia. Similarly, having the recommended dietary calcium intake was to the direction of protection from preeclampsia, even though statistically not significant. These associations also persisted and dietary calcium intake became statistically significant when imputed in the multivariate analysis (Table 3).

**Table 3:** Univariate and multivariate logistic regression for the factors associated with preeclampsia

Variable	Cases	Controls	*p*-value	cOR (95% C.I.)	*p*-value	aOR (95% C.I.)
**Parity**						
Nulliparous	22	17	0.28	1.62 (0.68–3.84)	0.95	1.04 (0.33–3.27)
Multiparous	20	25	*Ref*			
**Gestational age (weeks)**						
< 28	1	1	0.76	1.55 (0.09–26.01)	0.10	13.60 (0.63–294.62)
28–34.9	9	3	0.03	4.64 (1.13–19.04)	0.13	3.69 (0.70–19.65)
35–36.9	10	4	0.04	3.86 (1.08–13.86)	0.12	3.52 (0.72–17.30)
≥ 37	22	34	*Ref*			
**Ionized Calcium level (mmol/l)**						
< 1.16	29	9	0.00	8.1 (3.05–21.91)	0.00	7.51 (2.39–23.60)
≥ 1.16	13	33	*Ref*			
**Total serum calcium level (mmol/l)**						
< 2.1	29	15	0.00	4.02 (1.62–9.97)	0.045	3.10 (1.02–9.37)
≥ 2.1	13	27	*Ref*			
**Dietary calcium intake (gm/24 h)**						
< 1000	27	18	0.05	2.40 (1.00–5.78)	0.03	3.42 (1.10–10.72)
≥ 1000	15	24	*Ref*			

*cOR* Crude odds ratio, *aOR* Adjusted odds ratio, *95% C.I.* 95% confidence interval

When compared to pregnancies which were ≥ 37 weeks, having a preterm pregnancy increased the risk of preeclampsia on univariate logistic regression, but when gestational age was imputed into the multiple logistic regression model, it lost its statistical significance (Table 3).

On multivariate analysis, the odds of having preeclampsia was almost eight times higher among those participants with low ionized calcium level (OR 7.5, 95% CI 2.388–23.608) and three times higher in those with low total serum calcium level (OR 3.0, 95% CI 1.024–9.370). Low dietary calcium intake also showed statistically significant association with preeclampsia (OR 3.4, CI 1.092 -10.723). (Table 3) Age, parity, BMI, education, employment and income did not show any association with preeclampsia on univariate logistic regression.

On Pearson correlation, Serum ionized calcium level and total serum calcium level showed a positive correlation of moderate strength (*p* = 0.004, *r* = 0.307). But dietary calcium intake was not found to correlate with both total serum calcium level and ionized calcium level (Table 4).

**Table 4:** Correlation between total serum calcium, ionized calcium levels and dietary calcium intake

*Variable*	*Ionized calcium level, mmol/l*		*Dietary calcium amount, gm/day*	
	*P*	*r*	*P*	*r*
Total serum calcium level, mmol/l	0.004	0.307	0.352	0.103
Dietary calcium amount, gm/day	0.521	0.071		

*PP-*value, *r* correlation coefficient

Chi square test did not show significant association between dietary calcium intake and educational level, employment, income, BMI, parity, age group, gestational age, total and ionized serum calcium levels.

Severity of preeclampsia showed statistically significant association with total serum calcium level (OR 0.096, 95% CI 0.018–0.518), but not with ionized calcium level and dietary calcium intake.

## Discussion

In this study, the mean total serum calcium level in preeclamptic women was lower than the normal reference range and from the mean in the controls. This is in agreement with other studies done in Sudan, India, South-Korea, Japan, Iran and in other low, middle- and high-income countries [10–13]. The level of mean iodized calcium was also low in those women with preeclampsia. During pregnancy, the ionized calcium remains constant throughout gestation unlike the total calcium which is affected by hemodilution of pregnancy and disorders like malnutrition, nephrotic syndrome and liver diseases, hence making ionized calcium level by far the most important index to know the actual calcium status of the body [18].

This study offers an original contribution to the knowledge of possible relationship of ionized calcium level with preeclampsia.

The metabolism of calcium is complicated and its homeostasis depends on hormones, complex feedback loop and dietary calcium intake. But conflicting results are found with regard to the correlation of dietary calcium amount with serum levels of total and ionized calcium, as diet is not the only factor responsible for the serum levels. Our study showed no correlation between dietary calcium intake with either total serum calcium concentrations or ionized serum calcium level on Pearson correlation, but serum ionized calcium level and serum total calcium levels correlated positively *P* = 0.004. This lack of correlation of dietary calcium intake and total serum calcium level has also been demonstrated in a study in pregnant women in India and in the study done by Byrne FN et al. in non-pregnant population [17, 25]. Of note, ionized serum calcium concentrations were not analyzed, therefore it was difficult to compare.

In our study, the median dietary calcium intake assessed with 24-hour dietary recall was 704 mg/24 h (IQR 458–1183) among the pre-eclamptic women, and 1115 mg/24 h (IQR 753–1878) in the controls. The calcium intake in the preeclamptic women was lesser than RDA, which at least, is 1000 mg/day during pregnancy. There is no study to compare this result with other findings, but our data shows a better dietary calcium intake in pregnant women in general compared with the general population according to the national survey result, which estimated it to be 317.32 mg/day in women of child bearing age [26].

The multivariate logistic regression showed that low dietary calcium intake was found more in the preeclamptic women compared with the controls (OR 3.4, CI 1.092 -10.723). There are only few studies done to see whether low dietary calcium intake would be associated with preeclampsia observing conflicting results. A study in India showed association between low dietary calcium intake and preeclampsia and a study done in Canada did not [17].

On multivariate analysis, the odds of having preeclampsia was almost eight times higher among those participants with low ionized calcium level (OR 7.5, 95% CI 2.388–23.608) and three times higher in those with low total serum calcium level (OR 3.0, 95% CI 1.024–9.370). This association of low total serum calcium level with preeclampsia has been demonstrated in studies done in India, Sudan, South- Korea and Iran [10–13]. As has been discussed earlier, total calcium concentration is unreliable in predicting the calcium status of the body. In an attempt to evaluate a more reliable measure of body calcium, a study done in South Africa measured hair calcium level in patients with preeclampsia and normotensive women, and found no difference in hair calcium levels between the two groups [16]. We have not found any other study that tried to see the level of ionized calcium level with regard to its relation with preeclampsia to help us compare with our findings.

In this study, when total serum calcium level was normal, the odds of having severe preeclampsia was 91% less likely compared with those having a low total serum calcium level. This observation of low total serum calcium level was more evident in women with severe forms of preeclampsia, and was reported in a case–control study done in Bangalore, India [27].

The current study has strengths and limitations. This study is probably the first study in English language that observed the relation of preeclampsia with serum ionized calcium level, even though this fact made comparison of the current study with other studies difficult. Although possible efforts were made to reduce the risk of investigator administered bias by masking the data collectors and recall bias by recruiting only incident cases, both types of bias cannot be eliminated completely.

## Conclusion

This study showed significant association between low dietary calcium intake and low serum calcium levels with preeclampsia. Hence, this can be used as a supportive local evidence for the current context-specific recommendation of calcium supplementation in societies with low-dietary calcium consumption in an attempt to prevent preeclampsia. Therefore, implementation study should be considered in Ethiopia to look for the feasibility of the routine supplementation of calcium starting early during pregnancy. Until then, we recommend health education on the importance of adequate dietary consumption of calcium rich foods during pregnancy. Further large-scale study may be warranted to explain the complex interconnection between ionized calcium level and total serum calcium level with preeclampsia.

## Supplementary Information

**Additional file 1**.

**Additional file 2.**

## Data Availability

The datasets used and analyzed during the current study are available from the corresponding author on reasonable request.
