# Supplementary material for: Association of dietary calcium intake, total and ionized serum calcium levels with preeclampsia in Ethiopia
Source: BMC Pregnancy Childbirth. 2021 Jul 27;21:532. doi: 10.1186/s12884-021-04005-y (PMC8314521; doi:10.1186/s12884-021-04005-y)
Supplement: Supplementary file 1 — Additional file 1. [file 12884_2021_4005_MOESM1_ESM.docx]

ASSOCIATION OF DIETARY CALCIUM INTAKE AND SERUM CALCIUM LEVEL WITH PREECLAMPSIA IN ETHIOPIA: A CASE-CONTROL STUDY

Rahel D. Gebreyohannes^1^, Ahmed Abdella^2^, Wondimu Ayele^3^, Ahizechukwu C. Eke^4^

1. MD, Assistant Professor of Obstetrics and Gynecology, Addis Ababa University, College of Health Sciences, Addis Ababa, Ethiopia
2. MD, MPH, Associate Professor of Obstetrics and Gynecology, Addis Ababa University, College of Health Sciences, Addis Ababa, Ethiopia
3. Assistant professor, PhDc, Department of Preventive Medicine, Addis Ababa University, School of Public Health, Addis Ababa, Ethiopia
4. MD, MPH, Assistant Professor of Maternal Fetal Medicine, Division of Maternal Fetal Medicine, Department of Gynecology and Obstetrics, Johns Hopkins University School of Medicine, Baltimore, MD, USA

Corresponding Author:

Rahel Demissew Gebreyohannes,

Department of Obstetrics and Gynecology, Addis Ababa University, College of Health Sciences,

Addis Ababa, Ethiopia.

Email: raheldemissewgy@gmail.com

Phone number: +251911394570

Fax: +251115152753

Participant code_____________________ Date________

| 24 HOUR DIET RECALL | | | | | | | | | | | | | | | | | |
| --- | --- | --- | --- | --- | --- | --- | --- | --- | --- | --- | --- | --- | --- | --- | --- | --- | --- |
| What was the first thing you ate or drank yesterday? | | What was the amount of the food? | | | Type of measure | | | How many people consumed this? | | |  | | | How much of it did you consume? | | | How much was the left over? |
| ① |  |  | | |  | | | □ Not shared | | | ___ F: 13-15 yrs | | |  | | |  |
| ② |  |  | | |  | | | ___ M/F: <2 yrs | | | ___ M: 13-15 yrs | | |  | | |  |
| ③ |  |  | | |  | | | ___ M/F: 2-6 yrs | | | ___ F: 16-19 yrs | | |  | | |  |
| ④ |  |  | | |  | | | ___ M/F: 7-9 yrs | | | ___ M: 16-19 yrs | | |  | | |  |
| ⑤ |  |  | | |  | | | ___ F: 10-12 yrs | | | ___ F: ≥20 yrs | | |  | | |  |
| At what time of the day? | |  | | |  | | | ___ M: 10-12 yrs | | | ___M: ≥20 yrs | | |  | | |  |
|  | |  | | |  | | |  | | |  | | |  | | |  |
|  |  |  | | |  | | |  | | |  | | |  | | |  |
| Food type | | ① | | | ② | | | ③ | | | ④ | | | ⑤ | | |  |
| Where did you get it? | | home/purchased/gift | | | home/purchased/gift | | | home/purchased/gift | | | home/purchased/gift | | | home/purchased/gift | | |  |
| How was it prepared? | | raw/fermented/cooked | | | raw/fermented/cooked | | | raw/fermented/cooked | | | raw/fermented/cooked | | | raw/fermented/cooked | | |  |
| How much did this recipe make? | | ① | | | ② | | | ③ | | | ④ | | | ⑤ | | |  |
|  | |  | ingredient | Measur. |  | ingredient | Measur. |  | ingredient | Measur. |  | ingredient | Measur. |  | ingredient | Measur. |  |
|  | | 1. |  |  | 1. |  |  | 1. |  |  | 1. |  |  | 1. |  |  |  |
|  | | 2. |  |  | 2. |  |  | 2. |  |  | 2. |  |  | 2. |  |  |  |
|  | | 3. |  |  | 3. |  |  | 3. |  |  | 3. |  |  | 3. |  |  |  |
|  | | 4. |  |  | 4. |  |  | 4. |  |  | 4. |  |  | 4. |  |  |  |
|  | | 5. |  |  | 5. |  |  | 5. |  |  | 5. |  |  | 5. |  |  |  |
|  | |  |  |  |  |  |  |  |  |  |  |  |  |  |  |  |  |
|  | |  | | |  | | |  | | |  | | |  | | |  |
