# Supplementary material for: Association of dietary calcium intake, total and ionized serum calcium levels with preeclampsia in Ethiopia
Source: BMC Pregnancy Childbirth. 2021 Jul 27;21:532. doi: 10.1186/s12884-021-04005-y (PMC8314521; doi:10.1186/s12884-021-04005-y)
Supplement: Supplementary file 2 — Additional file 2. [file 12884_2021_4005_MOESM2_ESM.docx]

ASSOCIATION OF DIETARY CALCIUM INTAKE AND SERUM CALCIUM LEVEL WITH PREECLAMPSIA IN ETHIOPIA: A CASE-CONTROL STUDY

**Rahel D. Gebreyohannes^1^, Ahmed Abdella^2^, Wondimu Ayele^3^, Ahizechukwu C. Eke^4^**

1. MD, Assistant Professor of Obstetrics and Gynecology, Addis Ababa University, College of Health Sciences, Addis Ababa, Ethiopia
2. MD, MPH, Associate Professor of Obstetrics and Gynecology, Addis Ababa University, College of Health Sciences, Addis Ababa, Ethiopia
3. Assistant professor, PhDc, Department of Preventive Medicine, Addis Ababa University, School of Public Health, Addis Ababa, Ethiopia
4. MD, MPH, Assistant Professor of Maternal Fetal Medicine, Division of Maternal Fetal Medicine, Department of Gynecology and Obstetrics, Johns Hopkins University School of Medicine, Baltimore, MD, USA

**Corresponding Author:**

Rahel Demissew Gebreyohannes,

Department of Obstetrics and Gynecology, Addis Ababa University, College of Health Sciences,

Addis Ababa, Ethiopia.

Email: raheldemissewgy@gmail.com

Phone number: +251911394570

Fax: +251115152753

**Addis Ababa University**

**College of Health Sciences**

**Questionnaire**

**Participant code number: _____________________________**

**Hospital/ Health center: _________________________**

**Date (DD/MM/YY): ______________________ Time (local time) ____________**

**Phone number 1: ____________________________**

**Phone number 2: _____________________________**

**Participant card number: _________________________________________**

**Name of health center she is referred from ______________________________ (NR- not referred)**

| **PART 1: SOCIO-DEMOGRAPHIC CHARACTERISTICS OF THE PARTICIPANTS:** | | | | | | | | | | |
| --- | --- | --- | --- | --- | --- | --- | --- | --- | --- | --- |
|  |  | | | | | | | | | |
|  |  | | | | | | | | | |
|  |  | | | | | | | | | |
|  | Age: | _____________ in years   - Unknown | | | | | | | | |
|  | Address: | City __________________  Sub-city _______________  Woreda ________________ | | | | | | | | |
|  | Educational level: | - Illiterate (1) - Pre-school (2) - Primary (3) | | | | | | - Secondary (4) - University and above (5) | | |
|  | Occupation: | - Employed, specify __________________________ - Unemployed | | | | | | | | |
|  | Family’s monthly income: __________________________ | | | | | | | | | |
|  | Number of family members: __________________________ | | | | | | | | | |
|  |  | | | | | | | | | |
| **PART 2: OBSTETRIC PERFORMANCE** | | | | | | | | | | |
|  | | | | | | | | | | |
| 2.1 | Parity: ________________________ | | | | | | | | |  |
| 2.2 Year of previous pregnancies | | | 2.3 Outcome of pregnancies (Alive/ Dead) | | | | | | | |
|  | | |  | | | | | | | |
|  | | |  | | | | | | | |
|  | | |  | | | | | | | |
|  | | |  | | | | | | | |
|  | | |  | | | | | | | |
|  | | |  | | | | | | | |
|  | | |  | | | | | | | |
|  | | |  | | | | | | | |
|  |  | |  | | | | | | |  |
| **PART 3: OBSTETRIC CHARACTERISTICS** | | | | | | | | | |  |
|  | | | | | | | | | |  |
| 3.1 | **Gestational age:** | | | | - known - unknown | | | | | |
| 3.2 | **Diagnosis** | | | | _______________ | | | | | |
| 3.3 | **Gestational age in weeks:** | | | | | _______________ | | |  |  |
| 3.4 | **When was hypertension diagnosed?** | | | | | ­­­­­­­­­­­­­­­­­­­­­­­­­­­­­­­­­­­­­­­­­­­­­­­_______________weeks | | |  |  |
| 3.5 | **Severity feature/complications?** | | | | - Yes - No | | | | | |
| 3.6 | **What is the severity feature?** | | | | | |  | | | |
|  | - Symptoms (headache, blurring of vision, epigastric pain) (1) - Severe hypertension (SBP≥160, DBP≥110) (2) - Acute Kidney Injury (3) - HELLP syndrome (4) - Thrombocytopenia (5) - High liver enzymes (6) | | | - High bilirubin (7) - Hemolysis (8) - High LDH (9) - Eclampsia (10) - Pulmonary edema (11) - IUGR (12) - IUFD (13) - Abruptio placenta (14) | | | | | | |
| 3.7 | **If pregnancy is terminated in the previous six weeks, what is the indication?**  **Specify,** ________________________________________________________ | | | | | | | | | |
| 3.8  3.9 | **Height in meters**  **Pre-pregnancy Weight in KG** | | | | | | _______________________  _______________________ | | | |
| 3.10 | **Pre-pregnancy BMI in kg/m^2^** | | | | | | _______________________ | | | |
| 3.11  3.12 | **MUAC in centimeter**  **Current BMI in Kg/m^2^** | | | | | | _______________________  ^___________________________________^ | | | |
| 3.13 | **Blood pressure in mmHg** | | | | | | ____________ Time________ (LT) | | | |
| 3.14 | **Blood pressure after 4 hours** | | | | | | ____________ Time________(LT) | | | |
| 3.15 | **Urine protein level in grams/24hrs** | | | | | | _______________________ | | | |
| 3.16 | **Urine protein level from the dipstick measurement** | | | | | | _________________ (ND- not done) | | | |

**COMPLETE BLOOD COUNT**

3.17 WBC_______________

3.18 Hemoglobin__________

3.19 HCT_______________

3.20 Platelet____________

**LIVER ENZYMES**

3.21 AST/SGOT ___________

3.22 ALT/SGPT____________

3.23 LDH ______________

3.24 Bilirubin(total)____________

3.25 Bilirubin Direct___________

3.26 **OTHER INVESTIGATIONS**
